# Supplementary material for: An Explorative Qualitative Study of the Role of a Genetic Counsellor to Parents Receiving a Diagnosis After a Positive Newborn Bloodspot Screening
Source: Int J Neonatal Screen. 2025 Apr 28;11(2):32. doi: 10.3390/ijns11020032 (PMC12101340; doi:10.3390/ijns11020032)
Supplement: Supplementary file 1 [file IJNS-11-00032-s001.zip › IJNS-3532535-supplementary.pdf]

| | | |
|---|---|---|
| **Establishing Rapport** | • Did you get onto teams ok?<br>• How has your day been going? | |
| **Before recording the interview** | As participant for verbal consent to participate in the interview. | |
| **Component of Interview** | **Key Question** | **Prompts** |
| **1. Parents experiences of NBS: identifying the participants expectations of NBS prior to receiving a positive result for SMA or an IEM condition.** | Could you please share your experience with the NBS (blood test or 'heel prick test')? | - Do you remember being told about the blood test or 'heel prick test'?<br>- What do you remember?<br>- Did you have any expectation of the test prior to the results?<br>- Did you feel comfortable with the information which was given and what it would mean for your child? |
| **2. Experience of receiving a positive NBS result: assessing the psychosocial/emotional challenges** | What do you remember about receiving the newborn screening result?'<br><br>What were your feelings in the days and weeks after receiving the result?<br><br>What sorts of questions did you have?<br><br>Who did you have around you to ask?' | - How did you feel when you received the result?<br>- Who gave you the result?<br>- What happened next?<br>- Who did you tell about the result? (eliciting where they sought support – friends, family, GP etc) |
| **3. Understanding of the genetic condition found through NBS** | Now I am going to ask you a bit about the condition that your child has.<br><br>If you were telling a friend or health care professional, could you describe the condition which was found during NBS?<br><br>Do you recall the way the condition is inherited?<br><br>Would you feel comfortable to share your thoughts on future pregnancies?<br><br>Was there any information which you felt was missed in the session?<br><br>Were there any questions you had that you don't feel were answered? | - Do remember what the condition was called and how it affects your child?<br>- Did a health professional tell you how the condition was inherited?<br>- Have you and your partner considered future pregnancies?<br>- How do you feel about thinking of future pregnancies?<br>- Do you know how likely this is to happen in a future pregnancy?<br>- IF, you still had questions, how did you navigate the answers? |

| | | |
|---|---|---|
| **4. Adaptation to the positive NBS result** | How long has it been since you received the result?<br><br>What sorts of things have been helpful in the past X months since you found out?<br><br>What has been more difficult for you?<br><br>Do you have any suggestions that would help us to provide good care in the time after receiving the results? | - Do you feel anxious/concerned/worried about sharing the NBS result?<br>- Have these feelings changed for you? |

# Consent Form

***An exploratory newborn bloodspot screening pilot study: comparison of the genetic counselling experience of families with spinal muscular atrophy (SMA) and metabolic disorders***

*Main Study Contact Person:* Dr Kaustuv Bhattacharya, Metabolic Paediatrician, Sydney Children's Hospital Network **kaustuv.bhattacharya@health.nsw.gov.au**

*Declaration by Participant*

☐ I have read the Participant Information Sheet or someone has read it to me in a language that I understand.

☐ I understand the purposes, procedures and risks of the research project described in the Participant Information Sheet (PIS).

☐ I have had an opportunity to ask questions and I am satisfied with the answers I have received.

☐ I freely agree to participate in this research project as described and understand that I am free to withdraw at any time during the project without affecting my family's future health care

☐ I understand that I will be given a signed copy of this document to keep.

☐ I wish to receive a lay summary of the study findings via my email address, which is the email address I was invited to partake in the study with.

Name of Participant (please print): ____________________________________________________

Signature of Participant: _____________________________ Date: _______________________

*Under certain circumstances (see Note for Guidance on Good Clinical Practice CPMP/ICH/135/95 at 4.8.9) a witness* to informed consent is required.*

Name of Witness* to Participant Signature (please print): ___________________________________

Signature of Witness: __________________________________________ Date: ________________________

* The Witness is not to be the investigator, a member of the study team or their delegate. In the event that an interpreter is used, the interpreter may not act as a witness to the consent process. Witnesses must be over 18 years of age

# Participant Information Sheet

***An exploratory newborn bloodspot screening pilot study: comparison of the genetic counselling experience of families with spinal muscular atrophy (SMA) and metabolic disorders***

**Project Investigators**

*Principal Investigator*: Dr Kaustuv Bhattacharya, Metabolic Paediatrician, Sydney Children's Hospital Network **kaustuv.bhattacharya@health.nsw.gov.au**

Study Team: Samantha Sandelowsky, Masters of Genetic Counselling Student, University of Technology Sydney A/Prof Carolyn Ellaway, Clinical Geneticist, Sydney Children's Hospital Network Kirsten Boggs, Genetic Counsellor, Sydney Children Hospital Network Jacqui Russell, Clinical Nurse Practioner (Clinical Genetics) Sydney Children's Hospital Network

You are invited to take part in the research study titled "***An exploratory newborn bloodspot screening pilot study: comparison of the genetic counselling experience of families with spinal muscular atrophy and metabolic disorders***". This study will be conducted by Samantha Sandelowsky, a University of Technology Masters of Genetic Counselling student based across the Sydney Children's Hospital Network. This project will be conducted with Dr Kaustuv Bhattacharya (Metabolic Genetics), A/Prof Carolyn Ellaway (Metabolic Genetics), Jacqui Russell (Clinical Genetics) and Kirsten Boggs (Clinical Genetics).

***What is this study's purpose?***

The aim of this research project is to explore the experience for families who received a positive newborn screen (NBS) result for Spinal Muscular atrophy (SMA) or conditions of Inborn Errors of Metabolism (IEM). This study will explore the experience for families with a diagnosis of either condition and gain further insight into family's adaption to a genetic diagnosis and the information shared about the condition detected via NBS. Currently, genetic counselling is only offered to families who received a positive NBS result for SMA. This study will contribute to the fulfilment of Samantha Sandelowsky Masters of Genetic Counselling (Coursework) at the University of Technology Sydney.

***Why have I been invited to be involved in this research study?***

You live in either NSW or ACT and have a child who was screened positive via NBS for a metabolic condition or spinal muscular atrophy.

***Do I have to take part in this study?***

Your participation in this research project is completely voluntary. Your decision to take part or not take part will not affect your relationship with the staff of the Sydney Children Hospital Network. If you decide to take part in the study, you will be given a consent form to sign and given a copy to keep.

***What would participation in this study look like?*** This study involves a single online interview which will be contacted via Microsoft Teams. These interviews are semi-structured and will be between 30-

45 minutes in length. These interviews can be conducted with both parents together if available, or separately, whichever suits you and your family. The interviews will be conducted by the Masters of Genetic Counselling research student, Samantha Sandelowsky.

***What are the potential risks of taking part in this study?***
The experiences we are discussing in the interview may be challenging for you. There may be interview questions which you find upsetting or stressful. You can ask the interviewer for a break or to skip a question. You can also ask for the interview to be stopped at any time. If you feel upset or distressed from participating in the research project, with your consent, a senior member of the research team will follow up with you.

***What are the possible benefits of taking part?***
There will be no direct benefit for you from participating in this study. As this is a pilot study (initial study in this area), the research results may provide data to inform other studies in this area. We hope that individuals and families who receive a positive NBS result for a metabolic disorder will benefit from an improved understanding of their education and psychological needs at the time of receiving the result.

***What will happen to my data*?**
If you decide to sign the consent form, you are consenting to the research team collecting and using personal information for this study. Your information will ONLY be used for the purpose of this research study. To protect your privacy, the study team will anonymise the interview responses. The interviews and transcripts will be stored on the Sydney Children's Hospital Network secure drive. Electronic data data from this study will be stored on this drive for a period of 15 years. Any physical data (i.e. signed consent forms etc) will be stored in a securely locked filing cabinet in an office located in the genetic metabolic disorders services officers at The Children's Hospital Westmead.

***What will the results from this study used for?***
The findings from this research study will be used for a manuscript for a Genetic Counselling Masters project. The results from this project will be published and potentially presented at conferences for genetics and metabolic conditions. Information found from this study will be reported in a way that cannot identify you or your family. You can also indicate on the consent form if you wish to receive a summary of the study findings.

**Who has reviewed the research project?**
All research in Australia involving humans is reviewed by an independent group of people called a Human Research Ethics Committee (HREC). The ethical aspects of this research project have been approved by the Sydney Children Hospital Network HREC (Approval number 2022/ETH02031).

This project will be carried out according to the *National Statement on Ethical Conduct in Human Research 2007 (including all updates)*. This statement has been developed to protect the interests of people who agree to participate in human research studies.

**Further information and who to contact**

In accordance with relevant Australian and NSW privacy and other relevant laws, you have the right to request access to your child's information collected and stored by the study team. You also have the right to request that any information with which you and your child disagrees be corrected. Please contact the study team member named at the end of this document if you would like to access yours or your child's information.

**Main contact person**

| | |
|---|---|
| Name | *Dr Kaustuv Bhattacharya* |
| Position | Metabolic Paediatrician |
| Telephone | *02 98453654* |
| Email | **kaustuv.bhattacharya@health.nsw.gov.au** |

If you or your child has any complaints about any aspect of the project, the way it is being conducted or any questions about your child being a research participant in general, then you may contact:

**Reviewing HREC approving this research and HREC Executive Officer details**

| | |
|---|---|
| Reviewing HREC name | Sydney Children's Hospital Network Human Research Ethics Committee |
| Position | Executive Officer |
| Telephone | (02) 9845 1253 |
| Email | SCHN-Ethics@health.nsw.gov.au |

The conduct of this study at Sydney Children's Hospital Randwick and The Children's Hospital Westmead has been authorised by the Sydney Children's Hospital Network. Any person with concerns or complaints about the conduct of this trial at the site may also contact the Research Governance Officer on 02 9845 1253 and quote protocol number 2022/ETH02031.
